# Update on the temper study: targeted monitoring, prospective evaluation and refinement

**DOI:** 10.1186/1745-6215-14-S1-P138

**Published:** 2013-11-29

**Authors:** Sally Stenning, Nicola Joffe, Priyanka Batra, Catrin Jones, Carlos Diaz Montana, Nancy Tappenden, Sarah Meredith

**Affiliations:** 1 MRC Clinical Trials Unit Hub for Trials Methodology Research, London, UK

## Background

On-site monitoring is a high-cost process, particularly if used indiscriminately in a large multicentre clinical trial. Ideally, the focus should be on identification and resolution of serious problems in trial conduct affecting patient safety and/or data quality. Targeted monitoring, where trial data and conduct are scrutinised centrally, with pre-specified triggers for site visits, may be an efficient way to prioritise on-site monitoring. Though widely used, this approach has not been formally evaluated.

## Methods

With funding from CRUK, and using a prospective matched-pair design, we are evaluating targeted monitoring within several multicentre cancer trials being conducted by the MRC Clinical Trials Unit. Using the pre-specified triggers for each trial, sites prioritised for a site visit will be matched (on number of patients recruited and time since trial site opened) with one that would not be visited based on the normal monitoring strategy. Paired sites will be visited and a standard monitoring report completed. The primary analysis will compare the proportion of sites with at least one critical or major monitoring finding in triggered vs. matched sites. 84 visits (42 pairs) will be carried out; the study is powered to detect a 30% difference in the proportion of sites with critical/ major findings (80% power, 5% 2 sided-significance level). An important secondary analysis will evaluate individual triggers as prognostic factors for findings.

## Status

The study is on-going; progress including development of reports to help monitor triggers, and the practical challenges in embedding evaluative methodological studies in open trials will be presented.

